# Assessment of left atrial and left atrial appendage flow and stasis in atrial fibrillation

**DOI:** 10.1186/1532-429X-17-S1-M3

**Published:** 2015-02-03

**Authors:** Michael Markl, Daniel C Lee, Maria L Carr, Charles Foucar, Jason Ng, Susanne Schnell, James C Carr, Jeffrey J Goldberger

**Affiliations:** 1 Northwestern University, Chicago, IL, USA

## Background

Atrial fibrillation (AF) is associated with increased risk of stroke due to development of left atrial (LA) thrombus [1]. Thromboembolic risk is assessed by clinical risk scores (CHA_2_DS_2_-VASc). However, these scores have limited predictive value. Echocardiography studies have shown that physiologic factors such as decreased atrial blood flow and increased stasis (LA velocities <0.2m/s) are associated with thrombus formation in the left atrial appendage (LAA) and may thus be better predictors for stroke [2]. Currently available diagnostic tools such as transesophageal echocardiography (TEE), however, are limited as they do not completely assess the complex 3D LA blood flow and are invasive. We have recently shown that atrial 4D flow MRI can overcome these limitations and detect physiologic changes in LA flow in patients with AF, i.e. potentially different predisposition to atrial thrombogenesis. However, previous studies were limited by the lack of a systematic evaluation of differences in LA vs. LAA (site of thrombus formation) and intuitive visualization and quantification of LA and LAA stasis [3].

## Methods

**Figure 1:**
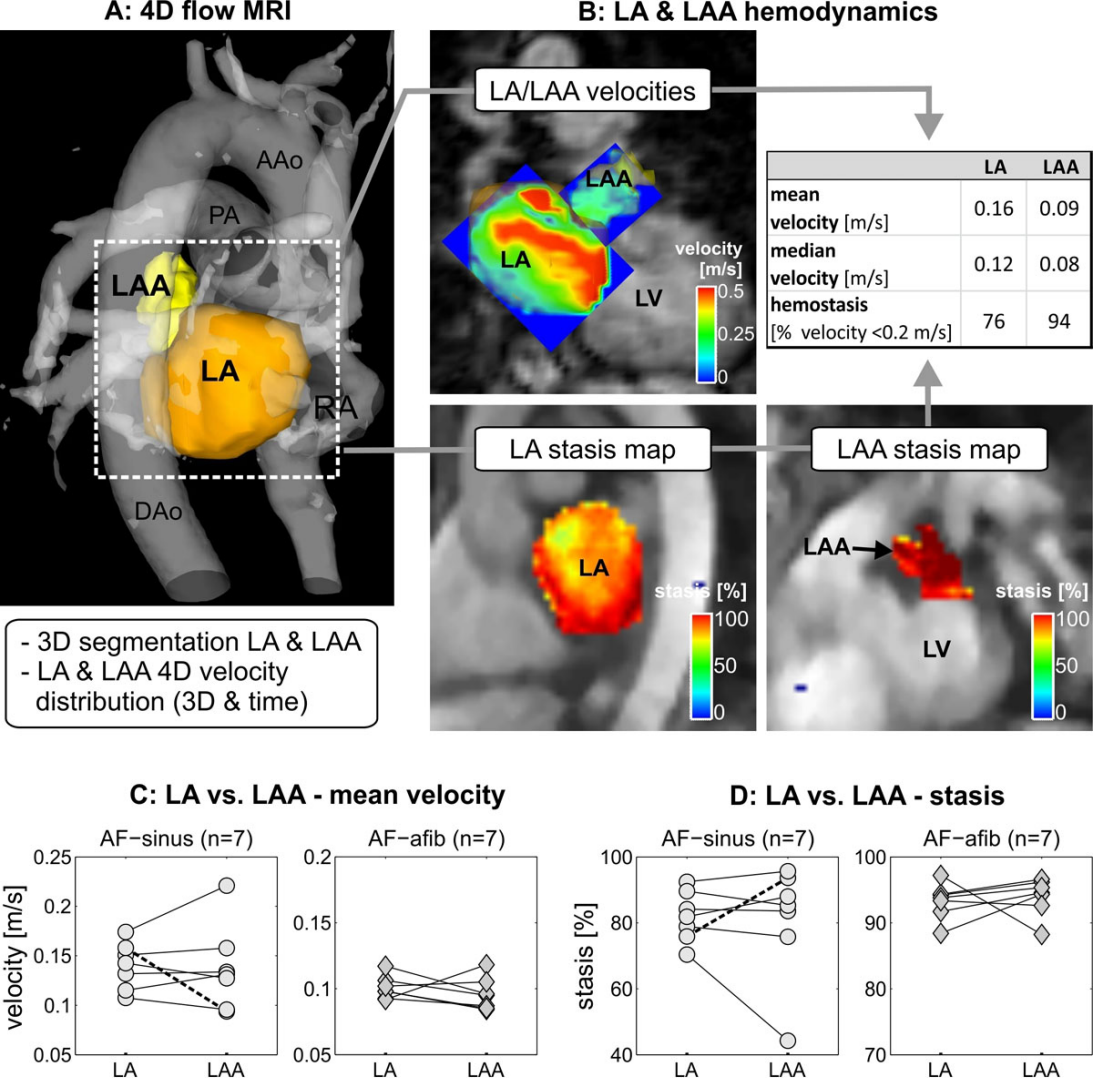
4D flow MRI (A) including 3D segmentation of the LA and LAA and calculation of LA and LAA stasis maps (B). Note the increased stasis in the LAA vs. LA in this AF patient. C, D: Mean velocity and relative stasis in the LA compared to the LAA in n14 AF patients. Dashed line = patient example in A,B.

4D flow MRI was employed to measure time-resolved 3D blood flow velocities in 14 AF patients: 7 in sinus rhythm (age=63±12years, AF-sinus), 7 with arrhythmia (age=70±9years, AF-afib). 3D segmentation of the LA and LAA (Fig. 1A) was used to isolate LA velocities and to quantify LA/LAA flow distributions. Stasis maps (Fig. 1B) were calculated to provide an intuitive visualization of stasis in the entire LA and LAA. For each LA/LAA voxel, the amount of flow stasis (in %) was calculated by determining the relative number of time frames with velocities <0.2m/s.

## Results

For an AF patient with CHA_2_DS_2_-VASc=1, Fig. 1A illustrates the strategy for assessing global and regional LA and LAA velocity and stasis (Fig.1B). In this seemingly low risk AF patient, flow quantification demonstrated considerably altered velocities and stasis in the LAA, the primary site of atrial thrombogenesis, compared to the LA. AF patients showed highly individualized differences between LA and LAA hemodynamics (Fig. 1C,D). Noticeably, AF patients in sinus rhythm at low CHA_2_DS_2_-VASc score (median=1) demonstrated increased LA vs. LAA variability (14±13% LA vs. LAA difference) compared to patients in persistent AF at higher risk (median CHA_2_DS_2_-VASc=4, 9±8% LA vs. LAA difference).

## Conclusions

Flow velocity and stasis measured in the LAA appear to be independent from the same measures in the LA. Thus, regional flow differences may be an important consideration for the assessment of thrombotic risk in patients with AF. In addition, our findings suggest that a seemingly low risk score, as defined by current clinical risk scores, may mask potentially relevant physiologic measures, such as elevated stasis in the LAA, the primary site of thrombogenesis.

## Funding

AHA (12GRNT12080032) and NIH-NHLBI (1R21HL113895).
